# Prevalence of Depression and Its Associated Factors among Orphan Children in Orphanages in Ilu Abba Bor Zone, South West Ethiopia

**DOI:** 10.1155/2018/6865085

**Published:** 2018-10-15

**Authors:** Gemechu Shiferaw, Lemi Bacha, Dereje Tsegaye

**Affiliations:** ^1^Department of Psychology, Faculty of Social Sciences, Mettu University, Mettu, Ethiopia; ^2^Department of Psychiatry, Faculty of Public Health and Medical Sciences, Mettu University, Mettu, Ethiopia; ^3^Department of Public Health, Faculty of Public Health and Medical Sciences, Mettu University, Mettu, Ethiopia

## Abstract

**Introduction:**

Orphans are the special group of children who are generally deprived and prone to develop psychiatric disorders even those reared in well-run institutions. These children and adolescents living as orphans or in stigmatized environments are vulnerable because of the loss of parent figures. The HIV/AIDS epidemic has contributed to a drastic increase in the number of orphans and vulnerable children and other causes in sub-Saharan Africa. However, little is known about the prevalence of depression and associated factors among orphanage children in areas such as Ethiopia.

**Objective:**

The aim of this study was to assess the prevalence of depression and its associated factors among orphans in Ilu Abba Bor Zone orphanages, 2016.

**Methods:**

An institutional based cross-sectional study was conducted among orphan children in orphanages at Mettu and Gore. A total of 220 orphans were included from the two orphanages and make the response rate of 98.2%. Pretested semistructured questionnaire was used for interviewing the study participants. The collected data were coded, entered into EPI-INFO 7.0. Software, and exported to SPSS version 20 for statistical analysis. The strength of association between variables was assessed using crude and Adjusted Odds Ratio by running logistic regression and the cut-off point for declaring statistical significance was P- value <0.05 or 95% confidence interval which does not contain the null value.

**Results:**

A total of 216 orphan children were interviewed with response rate of 98.2%. The overall prevalence of depression was 24.1%. The mean age of participants was 14.2 years ± 9.90 SDs and range from 11 to 17 years. Sex [Adjusted Odds Ratio = 3.29, 95% CI (1.41, 7.46)]; age [Adjusted Odds Ratio=2.09,95% CI (3.7; 5.01)]; duration of stay in foster care [Adjusted Odds Ratio= 2.08 (1.01; 8.33)]; previous physical abuse [Adjusted Odds Ratio= 3.1 (2.1; 5.06)]; having medical illness [Adjusted Odds Ratio=1.94,95% CI (2.01;3.56)]; orphan status [Adjusted Odds Ratio=2.5,95% CI (1.62; 3.56)]; and suicidal tendency [Adjusted Odds Ratio= 4.8 (3.41; 9.03)] were independent predictors of depression among orphans in orphanages.

**Conclusion and Recommendations:**

Prevalence of depression was high among orphans and this finding suggests that screening for depression and mental and psychological care should be integrated into routine health care provided to orphans and that there is a further need to establish preventive measures against depression.

## 1. Introduction

Childhood is a pivotal period for a child's overall development [1]. The survival and development of a child's optimal potential are disrupted if the family environment is jeopardized due to illness or death of the parents or guardians [2]. The loss of parents or guardians might also cause withdrawal, anxiety, and depression in adolescence.

Adverse childhood experiences (ACEs) are stressful events such as physical abuse, emotional abuse, sexual abuse, parental separation or divorce, neglect, and violence in the family [3]. ACEs could leave a long-lasting adverse impact on a child's mental development [4, 5].

Losing a parent and the bereavement that follows are difficult for children, and the effects may not manifest until many years afterwards [6]. Depression is a deep sadness with long-term, harmful effects on the health and development of the individual. When parents die, children not only miss their physical presence, but also miss the many positive things they gave them when they were alive, such as love, care, and protection. In many instances, orphans and vulnerable children have no one to share their grief with, and this can compound their sense of helplessness. Lack of support during the grieving process and inadequate help in adjusting to an environment without their parents may lead children to become depressed [7].

In addition, when orphans are placed with poorer households, anxiety about the future, including the prospect of not finishing school, may lead to depression [8]. Children entering foster care report more depressive symptoms and have higher prevalence of clinically significant depressive symptoms than children reared at home[9]. Researchers had found that orphaned children are more likely to suffer from internalizing problems, such as depression and anxiety [10, 11]. Another research had found orphans to be more depressed, more anxious, less optimistic about the future, and more likely to express anger feelings and have more disruptive behaviors compared to nonorphans [12].Children who are orphaned may suffer a loss of a former secure attachment, which has been suggested could put them at heightened risk of developing depression [13].

Children who are depressed are more often withdrawn from others and, so, may learn few social skills; they may have little energy for school work and so may fall behind academically, and their troubled emotions can strain relationships with caregivers, teachers, and fellow orphans who fail to recognize that they are depressed [14].

What is headache to many developing countries like Ethiopia is not only how to reduce the increasing number of orphan children but also how to deal with different psychological problems of these orphans. The findings of different studies indicated that orphan children are highly vulnerable to different psychological disorders such as PTSD, depression, anxiety disorder, and low self-esteem. Therefore, orphan children are highly in need of effective counseling to cope up with their problems [15].

Orphans are the special group of children who are generally deprived and prone to develop psychiatric disorders even those reared in well run-institutions [16, 17].

Children who grow up in orphanages or foster care usually have no social connections, and these children are at a disadvantage for completing high school, going to college, or getting job opportunities, all of which may be contributing factors in predisposing a child to psychopathology [10].

Yet there has been little research done on this area and this makes it difficult to find baseline data on how to evaluate and improve psychological support programs especially in this region and this study was conducted to assess the magnitude of depression and to identify factors associated with it and finally to indicate main intervention areas and make recommendations that will point out areas for further research by future investigators and provide insight into program planners and policymakers to review and change or strengthen existing care and support programs for children in the orphanages in the region and in the country as well.

## 2. Methods and Materials

### 2.1. Study Design and Setting

An institutional based cross-sectional study was conducted from May 1 to May 31, 2016, among orphans in orphanages in Mettu and Gore towns. Mettu is a capital town of I/A B Zone and is located 600 km away from Addis Ababa and 265 km from Jimma town. Gore is the town of Ale woreda and is 18 km away from Mettu to the South West direction. Mettu orphanage has 151 orphans while Gore orphanage cares for 73 orphans. These organizations aimed at providing various services such as accommodation, food, education, medical care, and counseling for orphan children who have lost their parents due to different causes.

### 2.2. Sample Size Determination and Sampling Techniques

All orphans in orphanages in the two centers were included in the study.

### 2.3. Data Collection Procedure

Data were collected using structured and pretested interview administered questionnaire that includes a tool that was used to evaluate the prevalence of depression. The Hospital Anxiety and Depression Scale (HADS) is initially developed to screen a self-assessment tool to identify anxiety and depression in patients. HADS could be used for patients, children, and adolescents, in primary care patients and in general population. And its Amharic version was validated in orphan adolescents in Addis Ababa [18]. Seven of the 14 items are for depression and seven are for anxiety with response ranges from 0 to 3 on a scale (3 indicates higher symptom frequencies). Score for each subscale ranges from 0 to 21 with score categorized as follows: normal 0-7, mild depression 8-10, moderate 11-14, and severe 15-21[19].

### 2.4. Data Quality Control

The questionnaire was designed and modified appropriately. It was translated to local language, Afaan Oromo, using the translation-back-translation method with two teams of translators to be understood by all participants. Training was given for data collectors and supervisors. Pretest was done before the start of actual data collection out of the study area and the results were not included in the main study. The data collectors were supervised daily and the filled questionnaire was checked daily by the supervisor and principal investigators. While problems were being faced, solutions were given by discussing with the supervisors and the data collectors.

### 2.5. Data Processing and Analysis

Data were checked for completeness and consistency. Then it was coded and entered into EPI-INFO version 7.0 and was exported to SPSS version 20.0 for analysis. Frequencies, percentages, mean crosstabulation, and odds ratio were calculated for different variables to describe the sample. Data were checked for normality distribution and outliers. Logistic regression and chi-square tests were used for comparison of the subjects with and without depression. P-value less than 0.05 was considered statistically significant association.

Bivariate and multivariate logistic regression analyses were used to identify independent predictors of depression among orphans in orphanages. The strength of the association was presented using odds ratio with 95% confidence interval (CI).

### 2.6. Ethical Consideration

Ethical clearance was obtained from the Institutional Review Board of Mettu University. The data collectors clearly explained the aims of the study to study participants and caregivers. Information was collected after obtaining verbal consent from each participant. The right was given to the study participants to refuse or discontinue participation at any time they want and the chance to ask anything about the study was given. For the purpose of anonymity participant's name was not used at the time of data collection and all other personal information kept entirely anonymous and confidentiality was assured throughout the study period. Data collectors signed that they could obtain verbal consent from the respondents. The investigator has commitment that findings will be used later to properly prevent and/or treat depression among orphans in orphanages.

## 3. Results

### 3.1. Basic Sociodemographic Characteristics of the Respondents

A total of 220 orphans participated in the study with a response rate of 98.2%. Of these, 113 (52.3%) were male orphans and 103 (47.7%) were female orphans. The mean age of the participant was 14.7 years ± 2.59 SDs and ranges from eleven to seventeen. Four-fifths (80.5%) were Oromo in Ethnicity. The study revealed that 126 (58.3%) of the participants were double orphaned and the remaining were single orphaned. As for the educational status of parents more than half (55.5%) attended primary and 29 (13.4%) do not have formal education (Table 1).

### 3.2. Basic Characteristics of Clinical Variables

Orphans with the duration of stay above two years were the predominant (53.2%). Participants who has additional medical illness were 32 (14.8%), and 29 (13.43%) had history of previous physical abuse. Thirty-nine (18.1%) had suicidal ideation.

### 3.3. Prevalence of Depressive Disorders

According to this study the overall prevalence of depression among orphans in orphanages was 24.1% and was slightly higher in females (27.2%) than in males (21.24%).

The prevalence was high among females, 28 (27.2%), age 15-17 years, 25 (32.9%), and those who live in Gore orphanages, 18 (26.1 %). Over a quarter of the respondents had five or more siblings, and the prevalence of depression was higher in this group (29.82%) than among respondents with fewer than five siblings. The main reason for nonresponse was repeatedly not being at institution at the time of data collection for medical cases.

The orphan institution is represented by more numbers of respondents; the prevalence of depression and depressive disorders is slightly higher in Gore than in Mettu orphanage. The prevalence is higher among 15-17 year olds but is the lowest among those with parents who are noneducated. Ninety-seven (44.9%) and 60 (27.78%) of the respondents' parents were in primary and secondary education, respectively; the prevalence of depression was the highest among respondents whose parents had no education and the lowest amongst those whose parents had higher education (Table 2).

### 3.4. Factors Associated with Depression among Orphans in Ilu Abba Bor Zone Orphanages

Both bivariate and multivariable logistic regression analyses were performed. In the bivariate logistic regression analysis, district, age, sex, number of siblings, duration of stay, counseling service, suicidal ideation, medical illness, orphan status, and physical abuse showed the association with the outcome variable.

In multivariable logistic regression analysis, age, sex, duration of stay, suicidal ideation, medical illness, orphan status, and physical abuse maintained association with the outcome variable.

Accordingly, sex was found to be significantly associated with depression; the odds of depression among female orphans were 3.29 times higher than the odds of depression among male orphans in orphanages [Adjusted Odds Ratio=3.29, 95% CI (1.41, 7.46)]. The odd of depression among respondents of age 15-17 years was 2.09 times higher than the odds of depression among age 11-14 years [Adjusted Odds Ratio=2.09, (3.7; 5.01)]. The odd of depression among double orphans was 2.5 times higher than the odds of depression among single orphans [Adjusted Odds Ratio= 2.5 (1.62; 3.56)].

The odd of depression among respondents with medical illness was 1.94 times higher than the odds of depression among those who have no medical illness [Adjusted Odds Ratio=1.94, 95% CI (2.01; 3.56)] and the odds of depression among those with suicidal ideation was 4.8 times higher than the odds of depression among orphans with no suicidal ideation [Adjusted Odds Ratio= 4.8 (3.41; 9.03)]. The odds of depression among respondents who stayed in orphanages for less than 2 years were 2.08 times higher compared with the odds of depression among those who were in orphanages for more than 2 years [Adjusted Odds Ratio = 2.08 (1.01; 8.33)]. The odd of depression among respondents with previous physical abuse was 3.1 times higher than the odds of depression among orphans with no history of physical abuse [Adjusted Odds Ratio = 3.1 (2.1; 5.06)] (Table 3).

## 4. Discussion

Mental health problem prevalence among children in difficult circumstances is higher than that of the samples taken from community or general population. For instance, the prevalence of mental illness among orphan children in six regions of Ethiopia was 31.3% of which depression was common [20].

Children entering foster care report more depressive symptoms and have higher prevalence of clinically significant depressive symptoms than children reared at home. [21]. This study showed that there was high prevalence of depression. According to this study the prevalence of depression among orphan children was found to be 24.1%. There have been no studies of depressions among orphans in orphanages in Ethiopia using a methodology similar to ours with which to directly compare our results. However the result of this study was consistent with the study conducted among orphans in Dakahlia and Sharkia governorate orphanages, in Egypt, which shows that 20% and 21% of orphans had depression, respectively. [21, 22] But it is lower than the study [23] performed on children admitted to orphanages in Gaza strip that reported the high prevalence of depression (41.5%). This difference may be due to recent loss of parents and being admitted to orphanages.

In this study, sex, orphans with medical illness, suicidal ideation, previous exposure to physical abuse, and lack of counseling services were independently significantly associated with orphan depression.

Sex was found to be significantly associated with depression. The odds of depression among female orphans were higher than the odds of depression among male orphans in orphanages. This finding is similar to a study in India, Visakhapatnam city, which showed that girls were more likely to be depressed than boys [24].

Depression was associated significantly with medical disorders in this study. The odd of depression among respondents with medical illness was 1.94 times higher than the odds of depression among those who have no medical illness. This finding is similar to the study in Cambodia that showed that factors such as physical health may affect mental health of OVC [25]. This finding is also consistent with other study findings that state that, in addition to depressed mood and irritability, children with depressive disorders also suffer from concentration difficulties, loss of appetite, or unspecific physical symptoms such as abdominal pain or headaches [16]. The prevalence rates are higher for children diagnosed with medical conditions [10, 11]. Depression rates have been reported at 14% among children and adolescents with cancer [15], 15% among youths with asthma [13], 23% among children with orthopedic procedures [14], and 26% among youths with burn injuries [15].This might be because prolonged illness might cause a sense of despair among the affected orphans as they had to cope with both the demands of the illness and their daily chores. On top of that, being very sick would affect the orphan's school attendance, and this in turn would affect the orphan's psychological well-being.

In this study, it was also found that those who got counseling services when they need have lower probability of being depressed. This is similar to studies done in Uganda, South Africa, and China [13, 17, 26]. Under various conditions, orphan children are highly exposed to multiple stressors that make their life more terrible and there is no or little formal counseling services available for them at a time when they are in need of them [8].

Children who have previous exposure to physical abuse were more likely to develop depressive symptoms than those who reported no physical abuse. This finding is also consistent with previous studies, 2012, in the UK which reported the interactive and cumulative effect of engagement in child labor and being physically abused heightened the risks of depressive symptoms from 38% to 66% and a study in South Africa stated that physical punishment of children using sticks and belts is in most African countries, virtually as a community norm [27]. Another finding of this study was that suicidal ideation is associated with depression and this finding is consistent with findings from Port Said city, Northern Egypt [28], and those from Uganda [29].

## 5. Limitation and Strength of the Study

The scope of the study is limited in terms of sample size and due to the fact that it is an institutional based cross-sectional design limiting the type of data collected. Despite the methodological limitations, the study revealed important information about the prevalence of depression and its associated factors among orphans in Ilu Abba Bor Zone. To date, this is the only study that explored depression and its associated factors among orphans in all orphanages of the Ilu Abba Bor Zone.

## 6. Conclusion and Recommendation

### 6.1. Conclusion

The prevalence of depression was high. Depression was significantly associated with female sex, duration of stay in orphanages, double orphans, those who do not get counseling services, medical illness, and suicidality.

### 6.2. Recommendation

Mental and psychological health care should be part of routinely provided care for orphans in the orphanages. Longitudinal and community based study should be recommended to identify the causal relationship of associated factors.

## Figures and Tables

**Table 1:** Distributions of respondents by sociodemographic characteristics in Ilu Abba Bor Zone, 2016.

**Characteristics**	**Category**	**Frequency**	**Percentage (**%**)**
**District**	Mettu	**151**	68.64
	Gore	69	31.36
**Age **	11–14	144	65.45
	15–17	76	34.55
**Gender**	Male	113	51.36
	Female	107	48.64
**Ethnicity**	Oromo	178	80.91
	Amhara	17	7.73
	Gurage	15	6.82
	Others	10	4.55
**Number of siblings**	0-2	73	33.18
	3-4	80	36.36
	5+	67	30.45
**Parent's education**	No formal education	27	12.27
	Elementary (Primary)	97	44.09
	Secondary	60	27.27
	Higher	36	16.36

**Table 2:** Distributions of sociodemographic characteristics and depression among orphans in orphanages of Ilu Abba Bor Zone, 2016.

**Characteristics**	**Category**	**Total (n)**	**Depression n (**%**)**
**District**	Mettu	**151**	34(22.5)
	Gore	69	18 (26.1 )
**Age **	11–14	144	25 (17.85)
	15–17	76	25 (32.9)
**Gender**	Male	113	24(21.24)
	Female	107	28 (27.2)
**Ethnicity**	Oromo	178	43(24.7)
	Amhara	17	4(23.53)
	Gurage	15	3(20)
	Others*∗*	10	2(20)
**Number of siblings**	0-2	73	18 (24.7)
	3-4	80	17 (19.8)
	5+	67	17 (29.8)
**Parent's education**	No formal education	27	6 (26.1)
	Elementary (Primary)	97	23 (23.7)
	Secondary	60	14 (23.3)
	Higher	36	9 (25)

*∗Tigree, Kefa, Daworo*

**Table 3:** Factors associated with depression among orphan children in orphanages of Ilu Abba Bor Zone, 2015.

***Characteristics***	***Category***	***Depression***		***Crude Odds Ratios (95***%*** CI)***	***Adjusted Odds Ratios*** *** (95***%*** CI)***	***P-value***
		***YES (***%**)**	***NO (***%**)**			
**District**	*Mettu*	34(22.5)	117(77.5)	**.82(0.20, 0.83)**	.45(.15, 1.29)	*0.33*
	*Gore*	18(26.1)	51(73.9)	**1**	1	
**Age group**	*11-14*	26(18.6)	114(81.4)	1	1	
	*15-17*	26(31.7)	56(68.3)	2.04(1.02,10.51)	2.09 (3.7; 5.01)	***0.026*** *∗*
***Sex***	*Male*	24(21.2)	89(78.8)	1	1	
	*Female*	28(26.2)	79(73.8)	**1.31 (1.21, 5.33)**	**3.29 (1.41, 7.46)**	**0.006** **∗**
***Number of siblings***	*0-2*	18 (24.7)	55(75.3)	**.96 (.15, .99)**	.82(.30, 2.22)	0.073
	*3-4*	17 (21.3)	63 (78.8)	**.79(.27, .84)**	.74(.28, 1.93)	
	*5+*	17(29.8)	50(70.2)	**1**	1	
***Duration of stay***	*<2 years*	25(25.0)	76 (75)	**1.12 (1.01,1.84)**	**2.08 (1.01; 8.33)**	0.001*∗*
	*>2years*	27(22.7)	92(77.3)	1	1	
**Counselling service **	No	35 (59.6)	24 (40.7)	12.35(11.21, 14.02)	5.36 (0.56, 6.29)	
	Yes	17 (10.6)	144 (89.4)	1	1	
**Suicidal ideation**	*No*	36(20.2)	142(79.8)	1	1	
	*Yes*	16(38.1)	26(61.9)	**2.43(1.61,4.20)**	**4.8(3.41; 9.03)**	0.002*∗*
***Medical illness***	*No*	31(16.0)	157(84.0)	**1**	1	
	*Yes*	21(66.0)	11(34.0)	9.67 (1.77, 10.05)	**6.94 (2.01,8.56)**	0.021*∗*
***Orphan status***	*Single*	23(26.0)	97(77.0)	1	1	
	*Double*	29 (29.0)	71(71.0.)	**1.72 (1.12, 10.21)**	**2.5 (1.62; 3.56)**	0.027*∗*
***Physical abuse***	*No*	17(12.0)	125(88)	1	1	
	*Yes*	35(45.0)	43(55)	5.98(1.62,6.92)	**3.1(2.1; 5.06)**	0.006*∗*

Note: *∗* p- value < 0.05.

## Data Availability

The data used to support the findings of this study are available from the corresponding author upon request.
